# Rapid shift in methane carbon isotopes suggests microbial emissions drove record high atmospheric methane growth in 2020–2022

**DOI:** 10.1073/pnas.2411212121

**Published:** 2024-10-21

**Authors:** Sylvia Englund Michel, Xin Lan, John Miller, Pieter Tans, J. Reid Clark, Hinrich Schaefer, Peter Sperlich, Gordon Brailsford, Shinji Morimoto, Heiko Moossen, Jianghanyang Li

**Affiliations:** ^a^Institute of Arctic and Alpine Research, University of Colorado, Boulder CO 80303; ^b^Department of Atmospheric and Oceanic Sciences, University of Colorado, Boulder CO 80303; ^c^Cooperative Institute for Research in Environmental Sciences, University of Colorado, Boulder CO 80309; ^d^Global Monitoring Laboratory, National Oceanic and Atmospheric Administration, Boulder CO 80305; ^e^Department of Tropospheric Chemistry, National Institute of Water and Atmospheric Research, Wellington 6021, NZ; ^f^Graduate School of Science, Tohoku University, Sendai 980-8578, Japan; ^g^Max Planck Institute for Biogeochemistry, Jena 07745, Germany

**Keywords:** methane, stable isotopes, greenhouse gases

## Abstract

The growth rate of the atmospheric abundance of methane (CH_4_) reached a record high of 15.4 ppb yr^−1^ between 2020 and 2022, but the mechanisms driving the accelerated CH_4_ growth have so far been unclear. In this work, we use measurements of the ^13^C:^12^C ratio of CH_4_ (expressed as *δ*^13^C_CH4_) from NOAA’s Global Greenhouse Gas Reference Network and a box model to investigate potential drivers for the rapid CH_4_ growth. These measurements show that the record-high CH_4_ growth in 2020–2022 was accompanied by a sharp decline in *δ*^13^C_CH4_, indicating that the increase in CH_4_ abundance was mainly driven by increased emissions from microbial sources such as wetlands, waste, and agriculture. We use our box model to reject increasing fossil fuel emissions or decreasing hydroxyl radical sink as the dominant driver for increasing global methane abundance.

Methane (CH_4_) is the second-most abundant anthropogenic greenhouse gas and has global warming potential (GWP) of 28 over 100 y (1); as a result, CH_4_ has consequential near-term radiative effects and is a prominent target for mitigation (2). Following a short pause in growth from 1999 to 2006, both the abundance and growth rate of atmospheric methane have been increasing (3). During 2020–2022, the observed CH_4_ growth rate reached a record high since NOAA measurements began in 1983, averaging 15.4 ± 0.6 ppb yr^−1^ (4). Understanding the mechanisms driving this accelerated growth is essential for predicting its future climate impact and providing scientific support for climate mitigation strategies (2).

The carbon isotopic composition of atmospheric CH_4_ (*δ*^13^C_CH4_) is a powerful tool for tracking the sources and sinks of atmospheric CH_4_. Different CH_4_ sources have distinctive *δ* ^13^C_CH4_ values: Microbial CH_4_ emissions (wetlands, livestock, landfills, etc.) have lower *δ*^13^C_CH4_ values (global mean of –62‰) than pyrogenic (biomass and biofuel burning, global mean of –24‰) and fossil fuel CH_4_ emissions (global mean of –45‰) (5). Various sinks of atmospheric CH_4_ also have distinctive isotopic effects. Therefore, combined observations of atmospheric CH_4_ mole fraction and *δ*^13^C_CH4_ can provide unique constraints on the changes of global CH_4_ sources and sinks during the post-2006 rapid CH_4_ growth.

The National Oceanic and Atmospheric Administration’s Global Monitoring Laboratory (NOAA/GML) has been carefully monitoring the global CH_4_ burden through the Global Greenhouse Gas Reference Network (GGGRN) for over four decades. The collaboration between NOAA/GML and the Institute of Arctic and Alpine Research (INSTAAR) at the University of Colorado Boulder has enabled *δ*^13^C_CH4_ measurements from the GGGRN since 1998, currently measuring weekly or biweekly from 22 globally distributed background sites (6). The dataset has been widely used for studying the evolution of global CH_4_ sources and sinks (7–9). Here, we report our most recent observations of atmospheric CH_4_ mole fractions and *δ*^13^C_CH4_ values through the end of 2022 and then use a box model to examine and quantify the contributions of potential drivers of the record-high CH_4_ growth rate.

## Results and Discussion

**Fig. 1.**
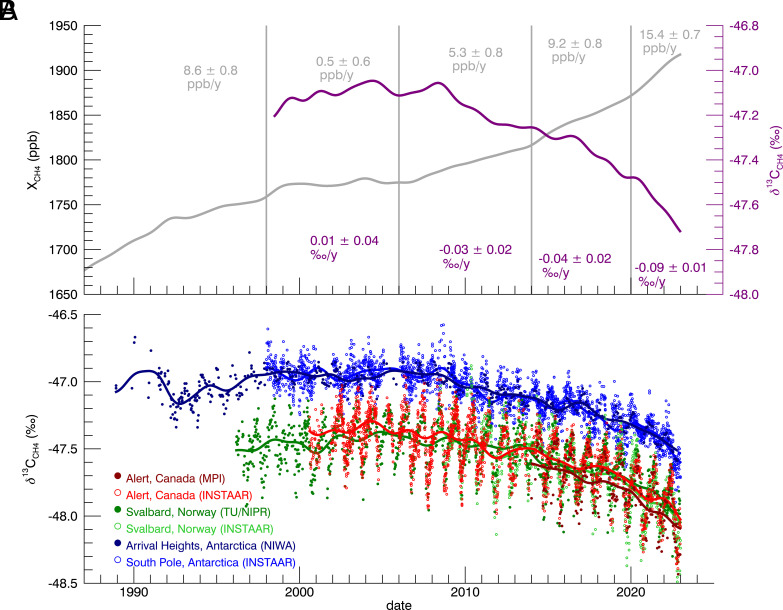
(*A*) Trend of globally averaged CH_4_ abundance (in gray) and *δ*^13^C_CH4_ (purple) from the NOAA/GML GGGRN. Mean growth rates of CH_4_ mole fraction and *δ*^13^C_CH4_ are shown for the following time periods: 1983–1998, 1999–2006, 2008–2014, 2014–2020, and 2020–2022. (*B*) Colocated *δ*^13^C_CH4_ measurements at Alert (Canada), Svalbard (Norway), and Antarctica by INSTAAR, NIWA, TU/NIPR, and MPI. Each dataset is fitted with a trend in the same color.

The global average methane growth rates in 2020, 2021, and 2022 reached record levels of 15.2 ± 0.45, 17.9 ± 0.45, and 13.1 ± 0.8 ppb yr^−1^, significantly higher than the average growth rates of 9.2 ppb yr^−1^ in 2014–2020, and 5.3 ppb yr^−1^ in 2008–2014 (Fig. 1*A*). Meanwhile, we observed the lowest global average *δ*^13^C_CH4_ in the observational record: –47.67 ± 0.01‰ in 2022. The global *δ*^13^C_CH4_ growth rate from 2020–2022 was –0.09 ± 0.01‰ yr^−1^, a much faster decrease than –0.04 ± 0.02‰ yr^−1^ in 2014–2020 and –0.03 ± 0.02‰ yr^−1^ in 2008–2014 (Fig. 1*A*).

The rapid decrease in *δ*^13^C_CH4_ in 2020–2022 is observed by multiple long-term monitoring programs: Max Planck Institute (MPI), National Institute of Water and Atmospheric Research (NIWA), and Tohoku University and National Institute of Polar Research (TU/NIPR, Fig. 1*B*), which have independent sampling schemes, analytical techniques, and data processing and quality protocols. These observations exhibit similar trends confirming the accelerated decreasing trend in atmospheric *δ*^13^C_CH4_ in 2020–2022 (*SI Appendix*).

**Fig. 2.**
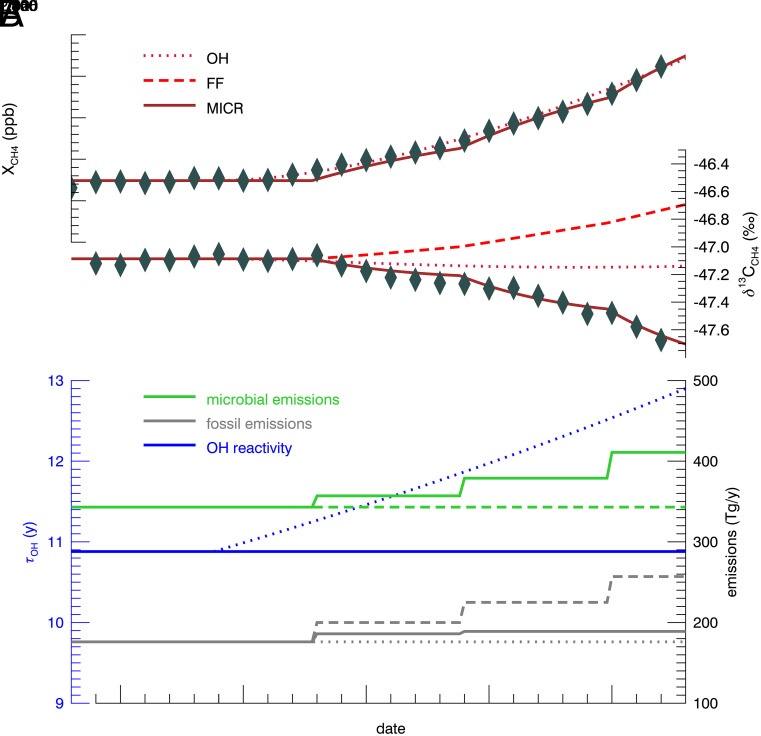
(*A*) Modeled response of CH_4_ mole fraction and *δ*^13^C_CH4_ due to different CH_4_ growth drivers. (*B*) Emissions and CH_4_ lifetime relative to OH for each scenario.

To investigate potential drivers for the rapid CH_4_ growth, we used a box model (10) to reconstruct the time series of global average CH_4_ mole fraction and *δ*^13^C_CH4_. Initial model emissions and sinks prior to 1999 are based on optimized values from a global 3-D inverse model (8) to allow the model to reach steady state with respect to CH_4_ mole fractions and *δ*^13^C_CH4_ during 1999 to 2006. We treated the time series as four segments (1999–2006, 2008–2014, 2014–2020, and 2020–2022), each with distinct CH_4_ and *δ*^13^C_CH4_ growth rates (Fig. 1*A*). We conducted different simulations to test the isotopic response to possible CH_4_ growth drivers (Fig. 2): 1) decreased OH in the troposphere (OH); 2) increased fossil-fuel emissions (FF); 3) increased microbial emission (MICR). In each simulation, we adjusted the flux of each source/sink category in each time segment to match the observed CH_4_ growth rate and then compared the resulting simulated atmospheric *δ*^13^C_CH4_ values to our observations.

Our model shows that only the MICR simulation displays a decrease in *δ*^13^C_CH4_. However, increasing only microbial emissions resulted in lower *δ*^13^C_CH4_ than the observations, so we also adjusted fossil fuel emissions to best fit both the observed CH_4_ mole fraction and *δ*^13^C_CH4_ (Fig. 2). Our best-fit result of the MICR simulation (*SI Appendix*) required an increase of microbial emissions over the steady state mean by 14 Tg yr^−1^ in 2008 with a concurrent increase in fossil emissions of 10 Tg yr^−1^; then in 2014, the microbial emissions increased by an additional 22 Tg yr^−1^, and fossil emissions increased by 3 Tg yr^−1^. These results are consistent with previous inverse modeling studies (8, 11, 12) that suggested approximately 85% of CH_4_ growth during 2007–2020 was due to increased microbial emissions. To capture the rapid growth in CH_4_ mole fraction and the decline of *δ*^13^C_CH4_ in 2020–2022, our model suggests an increase in microbial emissions of 32 Tg yr^−1^ in 2020 with no increase in fossil CH_4_ emissions required to match observations.

Decreases in biomass burning emissions between 10 to 30% over the past 2 decades (13, 14) could also explain some of the observed changes in *δ*^13^C_CH4_. Such decreases allow for more fossil emissions due to high *δ*^13^C_CH4_ from biomass burning. However, even considering the decreased biomass burning emissions, our model still suggests the post-2020 CH_4_ growth is almost entirely driven by increased microbial emissions (*SI Appendix*). Likewise, we modeled 1) a small increasing trend in OH number density (15), 2) an alternate OH fraction factor, and 3) a more negative *δ*^13^C_CH4_ value of fossil fuel emissions. In all scenarios, emission increases dominated by microbial sources are required to track both atmospheric CH_4_ and *δ*^13^C_CH4_ (*SI Appendix*). In this underconstrained problem, there are many ways to adjust model parameters to fit the model to the atmospheric data; however, all of the reasonable solutions require very large increases in microbial emissions. (An example of an unrealistic scenario would be an extreme case where biomass burning emissions decline to zero by 2020; only then do fossil fuel emission increases become comparable to those from microbial sources.)

Atmospheric *δ*^13^C_CH4_ does not allow us to differentiate between anthropogenic microbial sources (livestock, landfills) and natural ones (wetlands), so further study is necessary to investigate the potential climate feedback hypothesis (16). However, our box model suggests that microbial emissions played an even more significant role during 2020–2022 than in the years since 2008, which is in general agreement with studies that emphasize the key role of wetland emission increases to the recent global CH_4_ budget (11, 12, 17, 18).

## Materials and Methods

Atmospheric *δ*^13^C_CH4_ of background air samples collected from the GGGRN are measured using an Isotope Ratio Mass Spectrometer equipped with a custom-built extraction system which traps methane from whole air, focuses the sample, separates it from other carbon-containing compounds, combusts it to CO_2_, and measures it relative to a standard (6). Data extension and integration techniques were used to convert global measurements of CH_4_ and *δ*^13^C_CH4_ from the GGGRN into global averages and growth rates.

We used a two-box model with time steps of 0.2 y to investigate changes in sources and sinks that could match our observations of CH_4_ and *δ*^13^C_CH4_. The box model specifies CH_4_ emissions from microbial, fossil, and pyrogenic sources with prescribed *δ*^13^C values of –61.7‰, –44.8‰, and –24.3‰, respectively (5). Sinks include uptake by soil microbes, and oxidation by OH, Cl, and O(^1^D), all of which have associated kinetic isotope fractionation factors. The model was tuned to match observations from 1999–1996 and then adjusted to test the isotopic effects of different source/sink scenarios. More details are available in *SI Appendix*.

## Supplementary Material

Appendix 01 (PDF)

## Data Availability

Data have been deposited in NOAA Global Monitoring Laboratory Data Repository (https://doi.org/10.15138/JQEV-PF31) (19).
