# Supplementary material for: Rapid shift in methane carbon isotopes suggests microbial emissions drove record high atmospheric methane growth in 2020–2022
Source: Proc Natl Acad Sci U S A. 2024 Oct 21;121(44):e2411212121. doi: 10.1073/pnas.2411212121 (PMC11536133; doi:10.1073/pnas.2411212121)
Supplement: Supplementary file 1 — Appendix 01 (PDF) [file pnas.2411212121.sapp.pdf]

PNAS

Supporting Information for
Rapid shift in methane carbon isotopes suggests microbial emissions drove record high atmospheric methane growth in 2020-2022

Sylvia Englund Michel[1,2], Xin Lan[3,4], John Miller[4], Pieter Tans[1], J. Reid Clark[1], Hinrich Schaefer[5], Peter Sperlich[5], Gordon Brailsford[5], Shinji Morimoto[6], Heiko Moossen[7], Jianghanyang Li[1,2]

1 Institute of Arctic and Alpine Research, University of Colorado, Boulder CO, USA
2 Department of Atmospheric and Oceanic Sciences, University of Colorado, Boulder CO, USA
3 Cooperative Institute for Research in Environmental Sciences, University of Colorado, Boulder CO, USA
4 Global Monitoring Laboratory, National Oceanic and Atmospheric Administration, Boulder CO, USA
5 National Institute of Water and Atmospheric Research, Wellington, NZ
6 Tohoku University, Sendai, Japan
7 Max Planck Institute for Biogeochemistry, Jena, Germany

* Corresponding author: Sylvia Michel
Email: sylvia.michel@colorado.edu

This PDF file includes:

Supporting text
SI References

Supporting Information Text

Data

Methane mole fraction and $\delta^{13}C_{CH4}$ data used to calculate global averages come from NOAA Global Monitoring Laboratory's Global Greenhouse Gas Reference Network (1).

To measure carbon isotopes of CH_4, the INSTAAR Stable Isotope Lab uses the following steps in a "continuous flow" technique (which uses less air than dual inlet techniques): sample trapping onto a cold Hayesep-D (Restek) column; focusing on a cold PoraBond Q (Agilent) column; separation from other condensable compounds on a PoraBond Q column; combustion it at 1150 °C; separation from the potentially interfering krypton molecule on Porabond Q; measurement on an (GV, now Elementar) Isoprime Isotope Ratio Mass Spectrometer (IRMS). Each sample is measured relative to a CO_2 monitoring peak, and then samples are tied to the VPDB scale relative to CH_4 in air standards that are treated identically to the sample (2). Carbon isotope values are expressed as $\delta^{13}C_{CH4}$. the relative difference of $^{13}C/^{12}C$ to a standard, Vienna PeeDee Belemnite (VPDB).

Data integration and extension methods (3, 4) are used to calculate trends, growth rates, and global annual averages from NOAA/INSTAAR data that are considered representative of the marine boundary layer (as well as South Pole Station). Uncertainties are calculated using a Monte-Carlo approach that considers error introduced by analysis, the distribution of sites, and atmospheric variability. These three sources of error are added in quadrature at each time step. The uncertainties in $\delta^{13}C_{CH4}$ do not account for ties to VPDB.

Several labs contributed data to the study to show the robustness of the trends seen at NOAA GGGRN stations. The Max Planck Institute for Biogeochemistry (MPI) in Germany contributed data from Alert Station (ALT, same site as GGGRN ALT station: Nunavut, Canada, 82.45° N); Tohoku University/National Institute of Polar Research (TU/NIPR) in Japan contributed data from Ny Ålesund (Svalbard, Norway, 78.93° N), close to Zeppelin Station (ZEP: 78.90° N); and the National Institute of Water and Atmospheric Research (NIWA) in New Zealand contributed data from Arrival Heights, Antarctica (ARH: 77.83° S) which we compared to NOAA's South Pole station (SPO: 89.98° S).

Isotopic measurements made by MPI are similarly made using continuous flow (5–7) as are those by TU/NIPR (Morimoto et al., 2017). NIWA uses a dual inlet technique with offline methane extraction that requires substantially more air, but is inherently more precise due to longer, repeated measurement cycles (9, 10). All laboratories have different ties to VPDB (11). Inter-laboratory offsets were corrected for based on published (11) or direct comparisons between the labs and are 0.29, 0.13, and –0.18 ‰ for MPI, NIWA, and TU/NIPR respectively.

We assess the model and data over four periods which have distinct methane growth rates: 1999-2006, 2008-2014, 2014-2020, 2020-2022. We exclude 2007 because CH_4 and $\delta^{13}C_{CH4}$ have different inflection points. We calculated the growth rate at each site by each laboratory. SPO (INSTAAR) has the following growth rates for the four time periods: [0.006, –0.033, –0.031, –0.097, all in units of ‰ yr^{-1}] which are similar to ARH (NIWA): [0.001, –0.029, –0.034, –0.067]. Growth rates from INSTAAR data at ZEP [(not available), –0.022, –0.053, –0.089] are similar to those from TU/NIPR [0.005, –0.011, –0.036, –0.081]. INSTAAR data at ALT [–0.001, –0.016, –0.043, –0.094] are similar to those from MPI [(not available) –0.063, –0.035, –0.092]. Uncertainties on the slope were assessed with a simple Monte Carlo analysis using analytical reproducibility of 0.06 ‰ and are all less than $\pm$ 0.02 ‰ yr^{-1}.

Model Description

The model is a forward two-box model in which time step is 0.2 year, and the simulation runs for 500 years. Hemispheric exchange time is one year. Emissions are divided into microbial, fossil, and pyrogenic emission sources with $\delta^{13}C$ values of –61.7 ‰, –44.8 ‰, and –24.3 ‰. These values are taken from Sherwood et al (2021), a large database of isotopic source signatures that

combines measurements with estimates of flux; therefore, this signal number is representative of a globally averaged signature. The Sherwood et al value of fossil fuels, which have the largest range in $\delta^{13}C$, is supported by a recent database (12), which has an average value of –44.6 ‰ for fossil sources in Europe.

Sinks include destruction by OH (τ_{OH}=10.9) (13), tropospheric chlorine (τ_{Cl}=376.6) (14), and chemical destruction in the stratosphere (τ_{strat}=81.6) (15), all of which are first order. A zeroeth-order soil sink is also included: 17.7 Tg yr^{-1} in the northern hemisphere, and 6.9 Tg yr^{-1} in the southern hemisphere (16). Default fractionation factors for these are ε_{OH}=3.9 ‰, ε_{Cl}=61.9 ‰ (17), ε_{strat}=16.0 ‰ (15), and and ε_{soil}=24 ‰ (16). The proportion of emissions assumed to be in the Northern Hemisphere are 70% for microbial emissions, 90% for fossil fuels, and 50% for biomass burning.

We set the emissions from various categories and let it reach steady state during 2000-2006 period, and then initiate step changes in emissions at 2008, 2014, and 2020. Remarkably, simple step function changes in emissions allow us to fit our observations very well.

Model results

To meet the observations of CH_4 and $\delta^{13}C_{CH4}$, we set initial emissions values of 343 Tg yr^{-1} microbial, 176 Tg yr^{-1} fossil fuel, and 30 Tg yr^{-1} biomass burning. For the FF simulation, we increased fossil fuel CH_4 emissions to match the growth rate of CH_4 mole fraction during 2008-2014, 2014-2020, and 2020-2022, respectively. Our model suggests that fossil fuel CH_4 emissions need to increase by 24 Tg yr^{-1} in 2008, 25 Tg yr^{-1} in 2014, and 32 Tg yr^{-1} in 2020 to match the observed mole fraction of methane. As a result, the $\delta^{13}C_{CH4}$ values increased by 0.01 ‰ yr^{-1} during 2008-2014, 0.03 ‰ yr^{-1} during 2014-2020, and 0.04 ‰ yr^{-1} during 2020-2022.

The OH simulation increased the lifetime of CH_4 with respect to OH (τ_{OH}) to match the growth rate of CH_4 mole fractions during 2008-2014, 2014-2020, and 2020-2022. Our model suggests that increasing τ_{OH} by 0.10 yr yr^{-1} starting in 2004 could reproduce the observed CH_4 growth from 2008-2022. As a result, the $\delta^{13}C_{CH4}$ values increased by –0.006 ‰ yr^{-1} during 2008-2014, –0.002 ‰ yr^{-1} during 2014-2020, and 0.003 ‰ yr^{-1} during 2020-2022.

Our first MICR simulation increased only microbial CH_4 emissions to match the growth rate of CH_4 mole fraction during 2008-2014, 2014-2020, and 2020-2022. Similar to the FF simulation, our model suggests that microbial CH_4 emissions need to increase by 24 Tg yr^{-1} in 2008, 25 Tg yr^{-1} in 2014, and 32 Tg yr^{-1} in 2020. As a result, the $\delta^{13}C_{CH4}$ values decreased by –0.04 ‰ yr^{-1} during 2008-2014, –0.06 ‰ yr^{-1} during 2014-2020, and –0.08 ‰ yr^{-1} during 2020-2022.

Our best fit result of the MICR simulation, where the growth rate of $\delta^{13}C_{CH4}$ matched the model, required an increase of microbial emissions by 14 Tg yr^{-1} in 2008 with a concurrent increase in fossil emissions of 10 Tg yr^{-1}; then in 2014, the microbial emissions increased by an additional 22 Tg yr^{-1}, and fossil emissions increased by 3 Tg yr^{-1}. In 2020, microbial emissions needed to increase by 32 Tg yr^{-1} while there was no necessary increase in fossil CH_4 emissions.

In addition to comparing the growth rates, we also calculated the root mean square deviation (RMSD) to assess the fit of the model simulations to the data. Emissions from fossil fuels and microbial sources could vary by $\pm$ 2 Tg yr $^{-1}$ to maintain acceptable (less than 0.04 ‰ RMSD) fit to the data. Therefore, a modeled increase of up to 2 Tg yr^{-1} fossil emissions could also be an acceptable fit of our model to the observations from 2020-2022.

Sensitivity Tests

We tested the effect of using a more negative source signature for fossil fuels. If we used a $\delta^{13}C$ value of –46.5 ‰, the partitioning of emissions between microbial and fossil were quite different; however, the model still required the same ratio of microbial/fossil increases to match the trends in observations as the best MICR simulation over the entire period 2006-2022. We also investigated the potential impact of reduced biomass burning CH_4 emissions to our model results.

In the optimized MICR simulation, we reduced the biomass burning CH_4 emissions, then calculated the emission increases from both fossil fuel and microbial sources which best fit the observed CH_4 mole fractions and $\delta^{13}C_{CH4}$ values. Since the biomass burning CH_4 emissions are highly uncertain, we examined two scenarios: 1) ~10% reduction (from 30 Tg yr^{-1} to 27 Tg yr^{-1}) of biomass burning from 2008-2022 (18) and 2) ~30% reduction (from 30 Tg yr^{-1} to 21 Tg yr^{-1}) of biomass burning from 2008-2022 (19). At 10% reduction, our best fit result suggests an increase of microbial emissions by 12 Tg yr^{-1} in 2008, with a concurrent increase in fossil emissions of 17 Tg yr^{-1}; then in 2014, the microbial emissions increased by an additional 20 Tg yr^{-1}, and fossil emissions increased by 3 Tg yr^{-1}. In 2020, microbial emissions needed to increase by 32 Tg yr^{-1} while no increase in fossil CH_4 emission was required. At 30% reduction, our best fit result suggests an increase of microbial emissions by 7 Tg yr^{-1} in 2008, with a concurrent increase in fossil emissions of 26 Tg yr^{-1}; then in 2014, the microbial emissions increased by an additional 16 Tg yr^{-1}, and fossil emissions increased by 9 Tg yr^{-1}. In 2020, microbial emissions increased by 32 Tg yr^{-1} and no increase in fossil emissions was required. In both cases, even though the decrease in biomass burning allowed for more fossil increases, microbial emissions dominated the increase in 2020.

We tested the hypothesis of Zhao et al (20) where OH number density decreased from 2000-2010 by 0.3% yr^{-1}. With the increase in τ_{OH}, we were able to match the data by increasing microbial emissions 26 Tg yr^{-1} in 2008 and fossil emissions by 22 Tg yr^{-1}, increasing microbial emissions 12 Tg yr^{-1} in 2014 and fossil emissions by 1 Tg yr^{-1}, and increasing only microbial emissions by 32 Tg yr^{-1} in 2020. Though the microbial emissions needed in 2014 were somewhat less than other scenarios, the microbial emissions needed to match the data in 2020-2022 were the same. We also tested the scenario of Peng et al. (21) where OH number density increased by 1.6% between 2019-2020 (τ_{OH} decreased); this scenario had negligible effect on the modelled CH_4 and $\delta^{13}C_{CH4}$, and no adjustments to emissions were needed.

In all scenarios, the absolute values of the emissions are uncertain due to the uncertainties in the kinetic isotopic fractionation factors of the sink processes, but this has a much smaller influence on the changes in emissions or sinks needed to match the model to $\delta^{13}C_{CH4}$. We tested the influence of using the Cantrell et al. (22) value of the OH fractionation factor and found no effect on the ratio of fossil to microbial emissions needed to match the observations.

All data (including growth rates and comparison data) used in this study are available at https://doi.org/10.15138/JQEV-PF31.

1. X. Lan, K. W. Thoning, E. J. Dlugokencky, Trends in globally-averaged CH4, N2O, and SF6 determined from NOAA Global Monitoring Laboratory measurements. *https://doi.org/10.15138/P8XG-AA10* .

2. J. B. Miller, *et al.*, Development of analytical methods and measurements of 13C/12C in atmospheric CH4 from the NOAA Climate Monitoring and Diagnostics Laboratory Global Air Sampling Network. *Journal of Geophysical Research: Atmospheres* **107**, ACH-11 (2002).

3. K. A. Masarie, P. P. Tans, Extension and integration of atmospheric carbon dioxide data into a globally consistent measurement record. *Journal of Geophysical Research: Atmospheres* **100**, 11593–11610 (1995).

4. K. W. Thoning, P. P. Tans, W. D. Komhyr, Atmospheric carbon dioxide at Mauna Loa Observatory: 2. Analysis of the NOAA GMCC data, 1974–1985. *Journal of Geophysical Research: Atmospheres* **94**, 8549–8565 (1989).

5. A. Jordan, *et al.*, Atmospheric flask sampling program of MPI-BGC, version 2022.1. (2022).

6. A. M. H. R. M. B. W. H. M. Z. S. R. C. Jordan, “Atmospheric flask sampling program of MPI-BGC, version 2024.1” (2024).

7. W. A. Brand, M. Rothe, P. Sperlich, M. Strube, M. Wendeberg, Automated simultaneous measurement of the δ13C and δ2H values of methane and the δ13C and δ18O values of carbon dioxide in flask air samples using a new multi cryo-trap/gas chromatography/isotope ratio mass spectrometry system. *Rapid Communications in Mass Spectrometry* **30**, 1523–1539 (2016).

8. S. Morimoto, R. Fujita, S. Aoki, D. Goto, T. Nakazawa, Long-term variations of the mole fraction and carbon isotope ratio of atmospheric methane observed at Ny-Ålesund, Svalbard from 1996 to 2013. *Tellus B: Chemical and Physical Meteorology* **69**, 1380497 (2017).

9. H. Schaefer, *et al.*, A 21st-century shift from fossil-fuel to biogenic methane emissions indicated by 13CH4. *Science (1979)* **352**, 80–84 (2016).

10. D. C. Lowe, C. A. M. Brenninkmeijer, S. C. Tyler, E. J. Dlugkencky, Determination of the isotopic composition of atmospheric methane and its application in the Antarctic. *Journal of Geophysical Research: Atmospheres* **96**, 15455–15467 (1991).

11. T. Umezawa, *et al.*, Interlaboratory comparison of δ 13 C and δD measurements of atmospheric CH 4 for combined use of data sets from different laboratories. *Atmos Meas Tech* **11**, 1207–1231 (2018).

12. M. Menoud, *et al.*, New contributions of measurements in Europe to the global inventory of the stable isotopic composition of methane. *Earth Syst Sci Data* **14**, 4365–4386 (2022).

13. S. A. Montzka, *et al.*, New observational constraints for atmospheric hydroxyl on global and hemispheric scales. *Science (1979)* **288**, 500–503 (2000).

14. R. Hossaini, *et al.*, A global model of tropospheric chlorine chemistry: Organic versus inorganic sources and impact on methane oxidation. *Journal of Geophysical Research: Atmospheres* **121**, 14–271 (2016).

15. R. Hein, P. J. Crutzen, M. Heimann, An inverse modeling approach to investigate the global atmospheric methane cycle. *Global Biogeochem Cycles* **11**, 43–76 (1997).

16. S. L. King, P. D. Quay, J. M. Lansdown, The 13C/12C kinetic isotope effect for soil oxidation of methane at ambient atmospheric concentrations. *Journal of Geophysical Research: Atmospheres* **94**, 18273–18277 (1989).

17. G. Saueressig, *et al.*, Carbon 13 and D kinetic isotope effects in the reactions of CH4 with O (1 D) and OH: new laboratory measurements and their implications for the isotopic composition of stratospheric methane. *Journal of Geophysical Research: Atmospheres* **106**, 23127–23138 (2001).

18. M. Liu, L. Yang, A global fire emission dataset using the three-corner hat method (FiTCH). *Earth System Science Data Discussions* **2023**, 1–20 (2023).

19. J. R. Worden, *et al.*, Reduced biomass burning emissions reconcile conflicting estimates of the post-2006 atmospheric methane budget. *Nat Commun* **8**, 2227 (2017).

20. Y. Zhao, *et al.*, Influences of hydroxyl radicals (OH) on top-down estimates of the global and regional methane budgets. *Atmos Chem Phys* **20**, 9525–9546 (2020).

21. S. Peng, *et al.*, Wetland emission and atmospheric sink changes explain methane growth in 2020. *Nature* **612**, 477–482 (2022).

22. C. A. Cantrell, *et al.*, Carbon kinetic isotope effect in the oxidation of methane by the hydroxyl radical. *Journal of Geophysical Research: Atmospheres* **95**, 22455–22462 (1990).
